# The 5′-tail of antisense RNAII of pMV158 plays a critical role in binding to the target mRNA and in translation inhibition of *repB*

**DOI:** 10.3389/fgene.2015.00225

**Published:** 2015-06-30

**Authors:** Celeste López-Aguilar, Cristina Romero-López, Manuel Espinosa, Alfredo Berzal-Herranz, Gloria del Solar

**Affiliations:** ^1^Molecular Microbiology and Infection Biology Department, Centro de Investigaciones Biológicas (CIB-CSIC)Madrid, Spain; ^2^Molecular Biology Department, Instituto de Parasitología y Biomedicina López-Neyra (IPBLN-CSIC)Granada, Spain

**Keywords:** bacterial small non-coding RNAs, antisense RNA, target RNA, plasmid replication control, translation inhibition, RNA-RNA interaction

## Abstract

Rolling-circle replication of streptococcal plasmid pMV158 is controlled by the concerted action of two trans-acting elements, namely transcriptional repressor CopG and antisense RNAII, which inhibit expression of the *repB* gene encoding the replication initiator protein. The pMV158-encoded antisense RNAII exerts its activity of replication control by inhibiting translation of the essential *repB* gene. RNAII is the smallest and simplest among the characterized antisense RNAs involved in control of plasmid replication. Structure analysis of RNAII revealed that it folds into an 8-bp-long stem containing a 1-nt bulge and closed by a 6-nt apical loop. This hairpin is flanked by a 17-nt-long single-stranded 5′-tail and an 8-nt-long 3′-terminal U-rich stretch. Here, the 3′ and 5′ regions of the 5′-tail of RNAII are shown to play a critical role in the binding to the target mRNA and in the inhibition of *repB* translation, respectively. In contrast, the apical loop of the single hairpin of RNAII plays a rather secondary role and the upper stem region hardly contributes to the binding or inhibition processes. The entire 5′-tail is required for efficient inhibition of *repB* translation, though only the 8-nt-long region adjacent to the hairpin seems to be essential for rapid binding to the mRNA. These results show that a “kissing” interaction involving base-pairing between complementary hairpin loops in RNAII and mRNA is not critical for efficient RNA/RNA binding or *repB* translation inhibition. A singular binding mechanism is envisaged whereby initial pairing between complementary single-stranded regions in the antisense and sense RNAs progresses upwards into the corresponding hairpin stems to form the intermolecular duplex.

## Introduction

Prokaryotic antisense RNAs have been defined as small, diffusible, mostly untranslated and highly structured transcripts that pair to target RNAs to control their biological function (Wagner and Simons, 1994; Brantl, 2007). More specifically, antisense RNAs are referred to as the *cis*-encoded group of these regulatory RNAs, i.e., those transcribed from the DNA strand opposite their target RNA (Thomason and Storz, 2010). In the early 1980s, two natural antisense RNAs were independently found to control replication of plasmids ColE1 and R1 (Stougaard et al., 1981; Tomizawa et al., 1981) and shortly afterwards, the involvement of this class of transcripts in controlling gene expression in transposons and in the bacterial chromosome was also unveiled (Simons and Kleckner, 1983; Mizuno et al., 1984; Brantl, 2012). The widespread antisense RNA control of plasmid replication works by inhibiting the function of an essential RNA through a variety of modes of action (reviewed in Wagner and Simons, 1994; del Solar and Espinosa, 2000; Brantl, 2014). In some systems, the antisense RNA inhibits maturation of the replication primer (Masukata and Tomizawa, 1986). In most cases, however, the antisense RNA inhibits transcription and/or translation of the plasmid *rep* gene, which encodes the replication initiator (Rep) protein (Wagner and Simons, 1994; Chai and Winans, 2005). The antisense RNA systems of plasmid replication control also exhibit a variety of RNA/RNA binding pathways, which, in the few cases where the entire process has been dissected, initiate with a “kissing” step that involves reversible base-pairing between complementary hairpin loops. This initial pairing can progress into the upper part of the corresponding stems (Malmgren et al., 1997; Wagner and Brantl, 1998; Kolb et al., 2000). Subsequent formation of the RNA/RNA stable complex starts by base-pairing between a single-stranded region of the antisense RNA and its complementary accessible region in the target RNA (Tomizawa, 1985, 1990; Persson et al., 1990; Wagner and Simons, 1994). Mutational analysis has revealed the importance of the “kissing” interaction in the stable binding of the antisense/target RNA pair (Tomizawa, 1986; Wagner and Simons, 1994). In the streptococcal theta-replicating plasmid pIP501, efficient formation of a stable complex requires the presence of both the two 3′-terminal stem-loops and the unstructured central region of the antisense RNA (Brantl and Wagner, 1994). A two-step binding pathway was proposed for this antisense/sense RNA system, where initial “kissing” between two complementary loop pairs progresses through the entire stems to form a quasi-full-length duplex (Heidrich and Brantl, 2007). Although all well-characterized antisense systems of plasmid replication control initiate the antisense/target RNA binding through loop-loop pairing, linear-linear or loop-linear recognition mechanisms can also be envisaged. Actually, a loop-linear initial pairing similar to that previously reported for the R1 *hok/sok* killer system has been suggested for the antisense RNA system that controls replication of the *Rhizobium etli* p42d plasmid (Venkova-Canova et al., 2004; Cervantes-Rivera et al., 2010). In these two systems, the 5′ single-stranded tail of the antisense molecule pairs with a hairpin loop in the target mRNA to initiate RNA binding (Thisted et al., 1994; Franch et al., 1999; Cervantes-Rivera et al., 2010). When a loop-loop initial interaction scheme is used, a YUNR motif (*Y* = pyrimidine; *R* = purine) constituting a U-turn structure has been ubiquitously found in a recognition loop of either the sense or the antisense RNA. The singular configuration of the bases in the U-turn loop structure is thought to facilitate the initial base-pairing and the progression of the intermolecular helix, causing this motif to act as a general enhancer of RNA/RNA binding rates (Franch et al., 1999).

**Figure 1:**
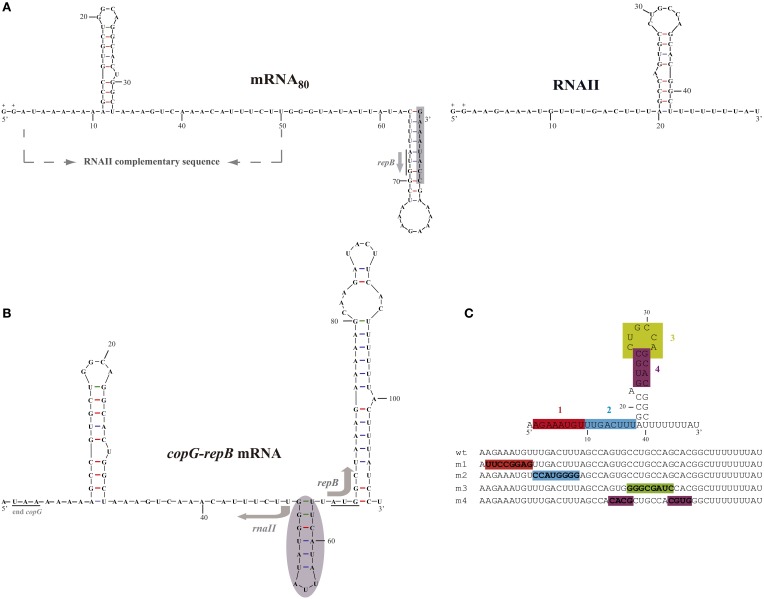
**Sequence and folding of the** ***in vivo*****- and** ***in vitro*****-synthesized antisense and target RNAs of the pMV158 replication control system. (A)** Observed secondary structure of *in vitro*-synthesized sense mRNA_80_ (left) and antisense RNAII (right). The 8-nt-long 3′ overextension observed in the mRNA_80_ transcript is shadowed. In the mRNA_80_ transcript the sequence complementary to antisense RNAII is shown, and the start codon of *repB* is underlined and indicated by an arrow. In both transcripts, the two extra Gs added at the 5′-end of the RNA sequence for efficient *in vitro* transcription (López-Aguilar and del Solar, 2013) are indicated (+). **(B)** Predicted structure of the intergenic region of the *copG-repB* mRNA. The *copG* stop codon and the *repB* start codon are underlined. The shadowed region upstream of the *repB* start codon has been shown to be involved in translation initiation of the gene (López-Aguilar et al., 2013). The start point and direction of transcription of *rnaII* and *repB* are indicated by arrows. **(C)** Antisense RNA mutants. Upper, RNAII regions whose sequence was changed to generate antisense RNA mutants m1 to m4 are shown in different colors. Lower, the same color code is used to display the sequence altered in each of the antisense mutants.

The promiscuous streptococcal rolling-circle-replicating plasmid pMV158 is the prototype of a growing family that so far includes about 70 members isolated from a variety of bacteria belonging to the *Firmicutes* or *Proteobacteria* phyla (Ruiz-Masó et al., 2015). Replicons of the pMV158 family are characterized by a high genetic economy that relies on the smallness of the genes encoding the replication initiator and the copy-number control elements, each among the smallest in its own class (del Solar and Espinosa, 2001; del Solar et al., 2002; Boer et al., 2009). A dual mechanism based on both a transcriptional repressor protein (CopG) and an antisense RNA (RNAII) has been shown to control initiation of replication of pMV158 (del Solar and Espinosa, 1992; del Solar et al., 1995; Hernández-Arriaga et al., 2009), and is also expected to control replication in most plasmids of the family. The *rnaII* gene, which encodes the 48-nt long RNAII, overlaps the intergenic region of the pMV158 *copG-repB* operon and is transcribed in the opposite direction to it. Therefore, RNAII is a countertranscript to the sense transcript, its entire nucleotide sequence being complementary to a *copG-repB* mRNA target region located immediately upstream of the atypical signals involved in translation initiation of the essential replication gene, *repB* (del Solar et al., 1997; López-Aguilar et al., 2013). In fact, RNAII-mediated control of pMV158 replication initiation has been shown to be exerted by inhibition of *repB* translation (del Solar et al., 1997). Recently, we have mapped the secondary structure of RNAII by chemical and enzymatic probing (López-Aguilar and del Solar, 2013). The experimentally proven secondary structure of RNAII, which matches the computational predictions, consists of a 17-nt A-U rich single-stranded 5′-tail followed by a typical rho-independent transcriptional terminator that is in turn composed of a G-C rich hairpin and a 3′ poly(U) tail (del Solar and Espinosa, 1992; López-Aguilar and del Solar, 2013; Figures 1A,C). *In vitro* transcription experiments showed that *Escherichia coli* RNA polymerase ends synthesis of RNA at this intrinsic terminator with an efficiency of ~90% (del Solar and Espinosa, 2001). Antisense RNAII fulfills all the features regarded as essential for an efficient plasmid replication control element, i.e., it is able to inhibit plasmid replication *in trans*, it has a short (1–2 min) half-life (Acebo et al., unpublished data), and it determines strong incompatibility against pMV158 (del Solar and Espinosa, 1992; del Solar et al., 1995). Derivatives of pMV158 that encode a mutant *copG-repB* mRNA lacking the hairpin complementary to that in RNAII were still susceptible to inhibition by the wild-type (wt) antisense RNA. These findings suggested that a “kissing” step was not strictly required for the RNA pairing mechanism in pMV158 (del Solar and Espinosa, 1992), and led to the proposal that *copG-repB* mRNA/RNAII binding initiates *via* a loop-linear pairing scheme (Franch et al., 1999). With a few exceptions, the existence of antisense RNAs controlling replication of plasmids of the pMV158 family has been assumed based on what had previously been shown for the prototype plasmid, and studies on the interaction with the cognate target mRNA are still missing. In pJB01 (a pMV158-family member), the existence of an antisense RNA controlling plasmid replication was proved, and the involvement of different regions of this RNA in inhibition of *rep* translation was studied by mutational analysis. Copy number inspection of the mutant plasmids suggested that the entire secondary structure of the antisense RNA was important for interaction with the target mRNA (Kim et al., 2008).

In the present work, we have analyzed the role of the different secondary structure elements of the pMV158-encoded antisense RNAII both in binding to the target mRNA and in inhibition of *repB* translation. The 3′-half of the 5′-tail has been found to be essential for efficient binding, whereas the 5′-terminal half, although contributing only moderately to mRNA binding, has a main role in inhibition of *repB* translation and hence in control of plasmid replication. Unlike most antisense RNA systems controlling plasmid replication, the apical loop of the single hairpin of RNAII has a rather secondary role in binding to the *copG-repB* mRNA, whereas the sequence of the upper stem is not required for either binding to the target mRNA or inhibition of *repB* expression.

## Materials and methods

### Bacterial strains, plasmids and oligonucleotides

*Streptococcus pneumoniae* 708 (*end*-1, *exo*-2, *trt*-1, *hex*-4, *malM*594) was employed as the host for all the *in vivo* assays. Pneumococcal cells were grown and transformed as described (Lacks, 1968). The plasmids used are listed in Table 1. Selection was applied by exposure to tetracycline (Tc; 1 μg/ml, or 0.5 μg/ml when specified) or chloramphenicol (Cm; 3 μg/ml) for pneumococcal cells containing pMV158-based or pC194-based replicons, respectively.

**Table 1:** **List of plasmids used in this work**.

**Plasmid**	**Size (bp)**	**Marker**	**Characteristics**	**References**
pLS1	4408	Tc^R^	Δ*mob*, ΔssoU derivative of pMV158	Lacks et al., 1986
pC194	2907	Cm^R^	Copy-up mutant of pC194 (~110 copies per chromosome equivalent in *S. pneumoniae*)	Ballester et al., 1990
pCGA9	3070	Cm^R^	*rnaII* gene of pMV158 cloned in pC194	del Solar et al., 1995
pCGA9-m1	3070	Cm^R^	*rnaII-m1* mutant gene cloned in pC194	*This work*
pCGA9-m2	3070	Cm^R^	*rnaII-m2* mutant gene cloned in pC194	*This work*
pCGA9-m3	3070	Cm^R^	*rnaII-m3* mutant gene cloned in pC194	*This work*
pCGA9-m4	3070	Cm^R^	*rnaII-m4* mutant gene cloned in pC194	*This work*
pALTER-T7-*copG-repB-cat*	7545	Cm^R^	Recombinant plasmid that carries *cop*G, *rep*B and *cat* genes of pJS3 cloned in pALTER-1 under the control of the ϕ10 promoter of phage T 7	del Solar et al., 1997

Oligonucleotides (oligos) used for amplification of DNA, *rnaII* mutagenesis, construction of DNA templates for *in vitro* transcription and oligo-RNA band-shift assays were chemically synthesized and then purified from 12% polyacrylamide (PAA) sequencing gels. All oligonucleotides employed in this work are listed in Supplementary Table 1.

### Plasmid DNA preparations and DNA manipulations

Total DNA preparations (crude extracts) from cells harboring plasmids were used to determine plasmid copy number as described (del Solar and Espinosa, 1992). Plasmid DNAs were prepared by the alkali denaturation method modified for *S. pneumoniae* (Stassi et al., 1981). DNA preparations were electrophoresed in 0.8–1% agarose gels. The nucleic acid bands were stained with ethidium bromide and visualized with the aid of a Gel-Doc documentation system. The Quantity-One software was used for the quantitative analysis of the fluorescence intensity of the different bands.

DNA fragments were purified from agarose gels by using the QIAquick Gel Extraction Kit (QIAGEN). DNA concentration was quantified using a NanoDrop™ spectrophotometer.

Enzymes for the modification of the DNA (restriction endonucleases, T4 DNA ligase and T4 polynucleotide kinase) as well as DNA polymerases were purchased from New England Biolabs and Fynnzymes, respectively, and used as specified by the suppliers.

### RNA extraction from pneumococcal cells

Total RNAs were prepared from exponentially growing pneumococcal cultures harboring the different pCGA9 plasmid variants (pCGA9, pCGA9-m1, -m2, -m3, and -m4), essentially as described (López et al., 1989).

### RNA secondary structure prediction

RNA structures were predicted by using the Mfold (3.2) program (Zuker, 2003)^1^.

### Directed mutagenesis of *rnaII* and construction of pCGA9 derivatives harboring the mutated genes

Mutant pCGA9 plasmids (pCGA9-m1, -m2, -m3, and -m4; Table 1) were generated by directed mutagenesis of the *rnaII* gene of pCGA9 (a pC194-based recombinant plasmid that carries the wt *rnaII* gene from pMV158; del Solar and Espinosa, 1992), followed by DNA amplification, digestion of the product with *Hae*III and *Pvu*II, and cloning of the appropriate fragment in pC194. Site-directed mutagenesis of *rnaII* was performed by the recombinant PCR method (Higuchi, 1990). Basically, two separate PCR were prepared by using as the primer pair either the forward oligo pC1800-1818 and one of the reverse mutagenic oligos (R-RNAII-m1, -m2, -m3, or -m4), or the reverse oligo pC2798-2780 and one of the forward mutagenic oligos (F-RNAII-m1, -m2, -m3 or -m4), and pCGA9 DNA as template. Each pair of overlapping primary PCR products were denatured and allowed to reanneal together, and the resulting heteroduplex with recessed 3′ ends was extended to generate a fragment that consists of the two primary products. This fragment was amplified using oligos pC1800-1818 and pC2798-2780, and subsequently subjected to restriction with *Hae*III and *Pvu*II. Next, the *Hae*III-*Pvu*II fragment was swapped into pC194, and the resulting recombinant plasmids were used to transform competent pneumococcal cells. The existence of pCGA9 derivatives containing the desired mutations in the *rnaII* gene was confirmed by automated DNA sequencing.

### Generation of DNA templates and *in vitro* synthesis of different RNA species

Double-stranded DNAs containing an improved promoter for T7 RNA polymerase (T7 RNAP)-mediated transcription (Moll et al., 2004; López-Aguilar and del Solar, 2013) followed by the sequence of the gene or gene fragment to be transcribed were used as templates to synthesize the wt and mutant antisense species, as well as the mRNA target (mRNA_80_) employed for the binding experiments. DNA templates encoding the different antisense RNA species were generated by extension of the common primer P-RNAII annealed to the following 83-mer oligos (Supplementary Table 1): RNAII (wt antisense RNA), RNAII-m1 (m1 mutant), RNAII-m2 (m2 mutant), RNAII-m3 (m3 mutant), or RNAII-m4 (m4 mutant). Then, the template DNAs were PCR-amplified using the primer pairs P-RNAII and P2-RNAII (for wt, m1, and m2 antisense RNAs), P-RNAII and P3-RNAII (for m3 antisense RNA), or P-RNAII and P4-RNAII (for the m4 mutant). The DNA template for *in vitro* synthesis of mRNA_80_ was generated by annealing and extension of oligos mRNA and mRNA-80 (Supplementary Table 1). The resulting double-stranded DNA was PCR-amplified using P-RNAII and mRNA-80R as the primer pair. The amplified DNA fragments were purified from PAA gels before being used as templates for synthesis of the various RNA species. The *in vitro* run-off transcription assays were performed using T7 RNAP from Roche, according to the manufacturer's protocol. The template DNAs were next removed by digestion with RNase-free DNase I (Roche). Samples were cleaned up by filtration through Sephadex G-25 columns (GE-Healthcare) and successive extraction with phenol/chloroform and chloroform, followed by ethanol precipitation. RNAs were quantified from their absorbance at 260 nm in a NanoDrop™ spectrophotometer.

The DNA template for T7 RNAP-mediated *in vitro* synthesis of the *copG-repB* mRNA employed in the experiments of *repB* translation inhibition was prepared by linearizing the recombinant plasmid pALTER-T7-*copG-repB-cat* (Table 1) at its single *Nco*I site located within the *cat* gene, as previously described (del Solar et al., 1997). The transcription assays were also carried out as described (del Solar et al., 1997). The resulting transcript specified full-length CopG and RepB proteins, and a truncated chloramphenicol acetyltransferase (T-Cat).

### 5′-end labeling of RNAs and oligonucleotides

*In vitro* synthesized antisense RNAs were dephosphorylated with alkaline phosphatase (Promega) following the manufacturer's indications. Then, the full-length RNA molecules were isolated from 12% PAA sequencing gels, extracted with phenol/chloroform and chloroform, and ethanol-precipitated, previous to the labeling of their 5′-end with T4 polynucleotide kinase (New England Biolabs) and [γ−^32^P] ATP. Labeled RNAs were re-isolated from 12% PAA sequencing gels as above.

The three 17-mer oligos (BS, BS-m3, and BS-m4, see Supplementary Table 1) employed in the oligo-RNA band-shift experiments were labeled at their 5′-end as described (del Solar et al., 1995).

### RNA-RNA binding assays

Previous to the binding reaction, the antisense and target RNAs were incubated separately at 65°C for 10 min and then at 37°C for 10 min, in order to obtain their native conformation. The binding mixtures (10 μl) contained 0.2 nM of any of the 5′-end-labeled antisense RNA variants and various concentrations (0–400 nM) of the unlabeled target RNA (mRNA_80_) in TMN buffer (100 mM NaCl, 20 mM Tris-OAc, 10 mM Mg(OAc)_2_. Binding was allowed for 30 min at 37°C, and terminated by adding an equal volume of loading buffer (glycerol 30%, tRNA 4%, xylene cyanol 0.4%, and bromophenol blue 0.4% in TMN buffer) and placing the mixture on ice. The samples were applied to non-denaturing 10% PAA gels. Gels were run at 300 V for 2 h.

Radioactive bands were visualized and quantified from fixed and dried gels using the storage phosphor technology, with the aid of a FLA-3000 (FUJIFILM) imaging system and the Quantity One software (Bio-Rad). The mRNA concentration required for half-maximal binding under the described experimental conditions (*K_d_*) was obtained with the Sigmaplot 9.0 software from data point fitting to the equation:

$$\text{y}={\text{B}}_{\text{max}}\text{​}\cdot \text{​x}/(K_{d}+\text{x})$$

where y is the fraction of complexed antisense RNA, B_max_ is the amplitude of the reaction, and x is the concentration of the target RNA.

### Time-course experiments of the RNA-RNA association

Standard binding mixtures containing 0.2 nM of the 5′-end-labeled antisense RNA and the indicated concentration, always in molar excess, of the unlabeled target RNA in TMN buffer were prepared as above and incubated at 37°C. Samples were withdrawn at different times and the reaction was stopped by diluting the samples into an equal volume of gel application buffer (94% formamide, 17 mM EDTA, 0.025% xylene cyanol, and 0.025% bromophenol blue) on ethanol-dry ice. Sample electrophoresis as well as visualization and quantification of the bands were performed as above. Least-squares analysis of the data from a log-linear plot of the fraction of free antisense RNA as a function of the incubation time yielded a straight line, at least until ~10% of the antisense RNA remained unbound. The slope of the curve was used to obtain the pseudo-first-order rate constant (b). The second-order rate constant (*k_a_*) was calculated as follows (Persson et al., 1988):

$$k_{a}=\text{b}/[\text{target RNA}]$$

where the concentration of the target RNA may be considered constant, due to the large molar excess of the mRNA relative to the antisense RNA.

### Antisense RNA-mediated inhibition of *in vitro repB* translation

*E. coli* S30 Extract System for Linear Templates (Promega) was employed to provide the host machinery needed for protein synthesis. Previous to the addition of the antisense and messenger RNAs, translation mixtures were prepared as directed by the manufacturers, maintained at 0°C, and treated with rifampicin (25 μg/ml) to prevent subsequent transcription from DNA traces that might be present in the mRNA preparation. Then, mRNA (2.5 μg) synthesized from pALTER-T7-*copG-repB-cat* (see above) and one of the antisense RNA variants (at a 20-Fold molar excess over the mRNA) were added, and the translation mixtures were incubated at 37°C for 90 min. *De novo*-synthesized proteins, labeled with [^35^S]-methionine, were separated on 15% SDS-PAA gels. Radioactivity in the bands was detected and quantified as described above. Values of the radioactivity in RepB were normalized with respect to those in CopG, whose synthesis is not regulated by the antisense RNA. Similar results were obtained when the values were normalized with respect to the levels of T-Cat.

### Incompatibility tests and copy number determinations

To test whether wt and mutant antisense RNAs exert incompatibility toward the pMV158 replicon, type I quantitative incompatibility tests (Nordström et al., 1980) were performed using as donor plasmids the pC194-based recombinants. Competent pneumococcal cultures (500 μl) harboring pLS1 (a pMV158 derivative, see Table 1) as the resident plasmid were transformed with 0.25 μg of DNA of the donor plasmid (pCGA9, pCGA9-m1, pCGA9-m2, pCGA9-m3, or pCGA9-m4). After allowing for phenotypic expression (70 min), cultures were induced with 0.5 μg/ml of Cm for 20 min, and transformants were selected in plates containing 3 μg/ml of Cm. Ten clones from each transformation were picked and grown in the presence of selective pressure for only the donor plasmid, and their total DNA content was analyzed by preparation of crude extracts and electrophoresis in agarose gels, as previously described (del Solar and Espinosa, 1992). The copy number of pLS1 in the heteroplasmid strains was compared with that in the homoplasmid strain. The percentage of pLS1-containing cells was also estimated at this stage (after ~37 generations without selective pressure for the resident plasmid) as the % of cells resistant to 0.5 μg/ml Tc, which is the lowest amount of antibiotic that provides selection (del Solar and Espinosa, 1992).

Whenever the presence *in trans* of a mutant antisense RNA did not result in clear incompatibility against pLS1 after ~37 generations of bacterial growth in the absence of Tc, three out of the transformant clones were grown for 60 more generations in the presence of only Cm, and the plasmid content as well as the percentage of Tc resistant (Tc^R^) cells was analyzed as above after each 10 generations.

### Oligo-RNA band-shifts assays

To determine the relative intracellular amount of the different antisense RNA variants, “oligo-RNA band-shifts” experiments were performed basically as described (del Solar et al., 1995). Total RNA (4 μg) from pneumococcal cultures harboring any of the recombinant pCGA9-type plasmids (wt or m1 to m4 mutants) was mixed with 0.25 pmol of the appropriate 5′-end-labeled oligo (BS for RNA extracted from pneumococcal cells containing pCGA9, pCGA9-m1 or –m2; BS-m3 for RNA obtained from cells containing pCGA9-m3; and BS-m4 for RNA extracted from cells harboring pCGA9-m4) in a final volume of 5 μl hybridization buffer (150 mM NaCl, 50 mM Tris-HCl pH 7.5, 1 mM EDTA). The mixture was heated at 75°C for 5 min and then slowly allowed to cool down to 42°C over a period of 120 min. Then the mixture was kept at 42°C for 30 more min before adding 1.5 μl of loading buffer (60% glicerol, 0.075% bromophenol blue, 0.25% xylene cyanol, 10 mM EDTA). Oligo-RNA hybrids were run on non-denaturing 15% PAA gels in Tris-acetate buffer (40 mM Tris-acetate, 1 mM EDTA) at 4°C, 110 V for 3 h. Labeled bands were visualized and quantified as described above. Each experiment was repeated at least twice for each of two RNA independent preparations. To estimate the amount of antisense RNA per μg of total RNA isolated from pneumococcal cells harboring a given pCGA9 plasmid variant, 0.025 and 0.0025 pmol of the corresponding *in vitro* synthesized antisense RNA variant were hybridized with the appropriate 5′-end-labeled oligo, and the samples were processed and analyzed in the same way as those containing the cellular RNA.

## Results

### Features of the antisense and sense RNAs synthesized *in vivo* and *in vitro*

The 5′-end of pMV158-encoded RNAII synthesized in pneumococcal cells has been determined by primer extension experiments (del Solar et al., 1997). Also, the 3′-end of this antisense RNA has been mapped on the basis of the increased size of the *in vitro* synthesized full-length transcript relative to run-off transcripts generated from the *rnaII* gene truncated at different positions (del Solar and Espinosa, 2001). Hence, antisense RNAII is a 48-nt-long transcript.

Chemical and enzymatic probing of *in vitro* synthesized RNAII (López-Aguilar and del Solar, 2013) has confirmed the previous computational predictions (del Solar and Espinosa, 1992) of the very simple secondary structure of this antisense RNA that comprises a single hairpin. RNAII consists of a 17-nt-long single-stranded 5′-tail followed by a hairpin structure (with a bulge and an apical loop) and a 3′-terminal U-stretch (Figure 1A). The hairpin and the U-stretch have been shown to be required *in vitro* for rho-independent transcription termination (del Solar and Espinosa, 2001). While performing this work, we observed that severe changes in the RNAII hairpin seemed also to impair transcriptional termination *in vivo*, leading to read-through transcription that, in the pCGA9-derivative plasmids encoding these extended RNAs, converged into the *cat* gene (see map of pCGA9 in del Solar and Espinosa, 1992) and prevented its expression and hence the Cm-resistance phenotype (not shown). Therefore, feasible selection of the pCGA9-type recombinants for Cm resistance implies that the cloned *rnaII* mutants encode an efficient intrinsic terminator.

In this work, wild-type RNAII and four antisense RNA mutants (m1, m2, m3, and m4) harboring 8-nt sequence substitutions in different elements of the RNAII secondary structure have been synthesized both *in vivo* and *in vitro* in order to compare their ability to bind to the target RNA and to inhibit translation of the essential *repB* gene. The mutated regions (Figure 1C) corresponded, respectively, to the 5′- (region-1, nt 2–9) and 3′- (region-2, nt 10–17) halves of the 5′-tail; to the hairpin loop (region-3, nt 26–33), and to the upper stem (region-4, nt 23–26 and 33–36). To change the sequence of region-4 without modifying the stability of the hairpin, the left and right arms of the upper stem were swapped, thus maintaining the base pairing (Figure 1C). All the antisense RNA mutants were predicted to keep a similar transcription terminator hairpin whose existence is also indicated by preliminary RNA structure probing experiments (not shown). Moreover, the intrinsic terminators of the antisense RNA mutants appear to be functional in pneumococcal cells, as inferred from the feasibility of cloning the corresponding genes in pC194 and selecting the recombinants for Cm resistance (see previous paragraph).

*Cis*-encoded antisense RNAII is complementary to the untranslated intergenic region of the bicistronic *copG-repB* mRNA (Figure 1B). The mRNA target is predicted to consist of secondary structure elements (a stem-loop flanked by single-stranded regions) of complementary sequence to their corresponding counterparts in antisense RNAII (Figure 1B). Previously, we produced *in vitro* two different targets of the antisense RNA (mRNA_60_ and mRNA_80_) by T7 RNAP-mediated run-off transcription of the intergenic region of the *copG-repB* operon (López-Aguilar and del Solar, 2013). Both target RNAs, which comprised the entire sequence complementary to antisense RNAII and lacked any intrinsic terminator, started at the position 5′ adjacent to the *copG* stop codon and spanned supposedly 60 nt (mRNA_60_) or 80 nt (mRNA_80_) on the *copG-repB* mRNA. However, run-off target products were longer-than-expected and contained 3′ extensions that arose most likely from the RNA-dependent polymerase activity of T7 RNAP and were involved in terminal hairpins (López-Aguilar and del Solar, 2013). Sequence and structure probing of both target RNAs showed that the mRNA_80_ mimicked quite well the structure predicted for the intergenic region of the *copG-repB* mRNA since it exhibited the sequences complementary to the 5′ and 3′ tails of RNAII in single-stranded conformation (Figures 1A,B). In mRNA_60_, by contrast, part of the sequence complementary to the RNAII region-1 was sequestered in the stem of the quite stable 3′-terminal hairpin (López-Aguilar and del Solar, 2013). The mRNA_80_ transcript was found to be 2–3 times more efficient in binding to RNAII than mRNA_60_ (López-Aguilar and del Solar, 2013). This different binding ability might be due to the distinct conformation of the region complementary to the 5′ terminus of antisense RNAII in both target transcripts, as it has been reported that the structural accessibility of the target RNA constitutes an important factor for antisense activity (Kittle et al., 1989; Wagner and Simons, 1994; Shao et al., 2006). The mRNA_80_ run-off transcript was chosen as the target RNA for analyzing the binding of the different antisense RNA species in view of the close resemblance between its structure and the structure predicted for the antisense RNA target region in the *copG-repB* mRNA.

### Region-2 of antisense RNAII plays a critical role in binding to the target mRNA

To know the contribution of the intermolecular pairing through different RNAII regions to the binding with the target RNA (mRNA_80_), we studied the efficiency of formation of RNA-RNA complexes that persist throughout electrophoresis on non-denaturing PAA gels. Two parameters were determined to analyze the efficiency of RNA-RNA binding: the concentration of free target RNA for half maximal binding (*K_d_*) and the association rate constant (*k_a_*).

**Figure 2:**
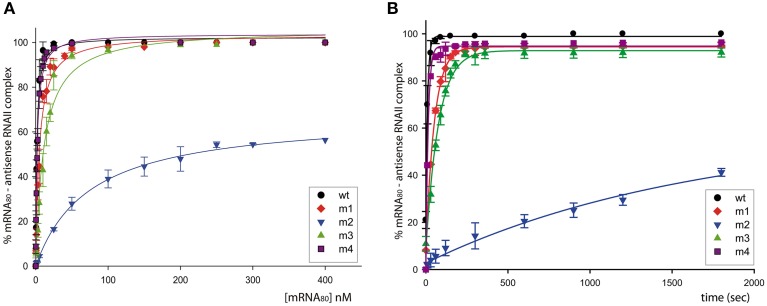
**Binding between the antisense RNA (wt or its variants) and the mRNA_80_ target. (A)** mRNA concentration dependence of the RNA-RNA binding. The binding mixtures were incubated for 30 min at 37°C. **(B)** Kinetic of the RNA-RNA association. Time-course experiments were performed in the presence of 150 nM of mRNA_80_.

RNA-RNA binding efficiency was most impaired by substitution of the RNAII region-2 (antisense RNA m2), which resulted in a 45-Fold increase in the concentration of free mRNA for half-maximal binding, and a 200-Fold decrease in the association rate constant (Figure 2 and Table 2). In contrast, substitution of region-1 (m1 mutant) or region-3 (m3 mutant) of RNAII caused only a 3.5- or 5.5-Fold increase, respectively, in the concentration of target RNA required for half-maximal binding, accompanied by a similar decrease in the association rate constant. Finally, the efficiency of binding was only slightly impaired in the antisense RNA m4, which has an altered sequence in region-4 (see Figure 2 and Table 2). These results show that the single-stranded regions of RNAII (regions 1, 2, and 3) contribute significantly to the binding to the target RNA, whereas the upper stem of the hairpin does not. In addition, region-2 is shown to have the greatest contribution.

**Table 2:** **Efficiency of binding of the different antisense RNA variants to mRNA_80_**.

**mRNA_80_**					
**Antisense RNA**	**Binding rate constant (*k_a_*) (10^5^ M^−1^s^−1^)**	**Relative *k_a_***	1/K^*^_d_ (nM^−1^)	**B_max_ ± s.d. (%)**	**Relative 1/*K_d_***
wt	3.75 ± 0.50	1	0.56 ± 0.05	102.39 ± 0.68	1
m1	1.08 ± 0.11	0.29	0.17 ± 0.01	103.56 ± 0.64	0.30
m2	0.02 ± 0.001	0.005	0.013 ± 0.001	67.58 ± 1.46	0.023
m3	0.66 ± 0.09	0.18	0.10 ± 0.01	106.26 ± 1.19	0.18
m4	2.29 ± 0.06	0.61	0.43 ± 0.02	103.89 ± 0.62	0.77

*Values are the mean of at least three independent assays*.

* *K_d_, concentration of free mRNA for half-maximal binding*.

### The entire 5′-tail of antisense RNAII is required for efficient inhibition of *in vitro repB* translation

**Figure 3:**
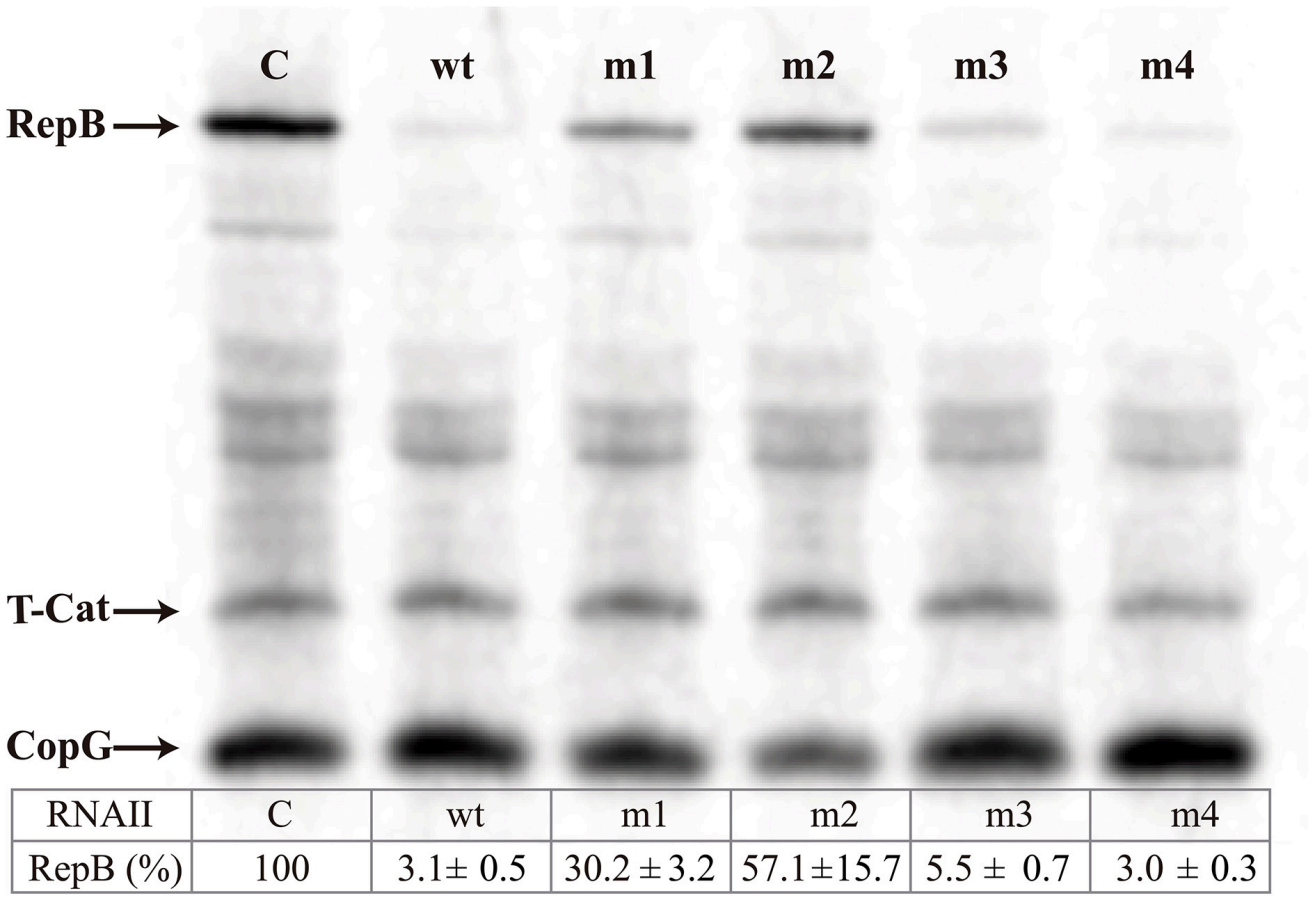
**Specific inhibition of RepB synthesis by antisense RNA variants**. *In vitro* translation of the *copG-repB*-truncated *cat* mRNA synthesized *in vitro* was performed either in the absence (C) or in the presence of the indicated antisense RNA variants. The table below shows the percentage of residual RepB synthesis. The numbers indicate the mean and standard deviation values obtained from at least four independent experiments.

The ability of the antisense RNA variants to inhibit translation of *repB* from an *in vitro* synthesized *copG-repB*-truncated *cat* mRNA was tested in *E. coli* S30 extracts. Quantification of the results (inserted Table in Figure 3) shows the percentage of RepB synthesized in the presence of the different antisense RNA variants, relative to the amount of *repB* translation in the absence of antisense RNA. The highest inhibitory activity was exhibited by the wt and m4 mutant antisense RNAs, which reduced *repB* translation to ~3%. Substitution of the RNAII hairpin loop (m3 mutant) only slightly impaired the inhibitory ability of the antisense RNA. In contrast, significant levels of RepB were synthesized in the presence of the m1 or m2 mutant RNAs, indicating that substitution of region-1 or, particularly, of region-2 resulted in a severe decrease of the inhibitory activity of the antisense RNA (Figure 3).

### The entire 5′-tail of antisense RNAII is required for efficient inhibition of pMV158 replication *in vivo*

**Figure 4:**
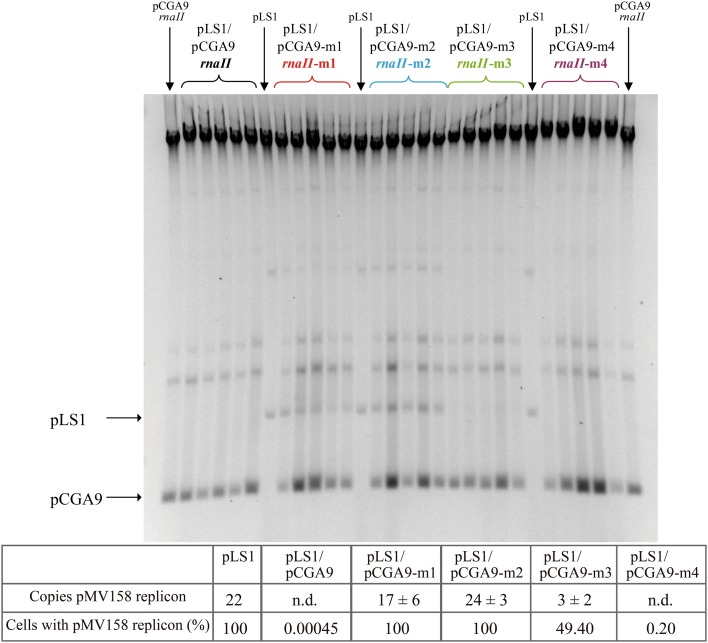
***In vivo*** **inhibition of the pMV158 replicon in the presence of the antisense RNA variants**. Incompatibility tests were performed as indicated in Materials and Methods. Pneumococcal cultures harboring plasmid pLS1, which contains the pMV158 replicon, were transformed with the indicated pCGA9 recombinant plasmid variants. The gel shows the total DNA content of several transformant clones. Homoplasmid strains harboring pLS1 or pCGA9 were used as controls. Supercoiled monomeric forms of both plasmids are indicated in the gel. The table below shows the average copy number of pLS1 and the % of cells retaining this plasmid, as estimated from at least 10 independent colonies. n.d.: not detectable.

Incompatibility tests were performed to analyze the effect of the different antisense RNA variants on the replication of pLS1, a plasmid derivative carrying the pMV158 replicon (Lacks et al., 1986). Genes encoding the RNAII variants (wt, m1, m2, m3, and m4) were cloned in pC194, a plasmid that has ~100 copies per chromosome equivalent in *S. pneumoniae* (Table 1) and is totally compatible with pLS1 (del Solar and Espinosa, 1992; del Solar et al., 1995). The resulting recombinant pCGA9 variants (see Materials and Methods) were transformed separately into competent pneumococcal cells containing pLS1. Ten individual clones from each transformation were grown for ~37 generations in the absence of selection for the resident plasmid, and the percentage of pLS1-containing cells as well as the average pLS1 copy number were then determined in all of them. *In vivo* replication of pLS1 was most impaired in the presence of wt RNAII synthesized from pCGA9: at the time of the analysis, only 0.5 × 10^−3^% of the cells contained pLS1 as an average (values ranged from <0.001 × 10^−3^ to 6.5 × 10^−3^% in the different colonies), and the DNA of this plasmid could not be detected in any of the clones (Figure 4). Antisense RNA m4 encoded by pCGA9-m4 also produced a strong incompatibility against the pMV158 replicon, which resulted in an average 0.20% (with values ranging from <0.001 × 10^−3^% to 0.95% in the different colonies) of cells containing the resident plasmid after ~37 generations, and again no detectable pLS1 DNA in the bacterial extracts (Figure 4). This indicates that pairing between RNAII and *copG-repB* mRNA through the bases located in the upper stems of the complementary hairpins is not required for efficient inhibition of pLS1 replication. RNA m3 still inhibited the pMV158 replicon though with a severely reduced efficiency compared to the wt RNAII, since at the time of analysis, plasmid pLS1 was present in about 50% (the percentage ranged from 2 to 76% in the different clones) of the cells containing pCGA9-m3, and its average copy number was estimated to be ~3 (Figure 4). Therefore, preventing formation of a “kissing complex” does not abolish the ability of the antisense RNA to inhibit *in vivo* pMV158 replication. In contrast, the m1 and m2 antisense RNA mutants lacked the *in vivo* inhibitory ability, so that plasmid pLS1 maintained the same copy number as in the homoplasmid strain in virtually all cells containing pCGA9-m1 or pCGA9-m2 (Figure 4). Total segregational stability of pLS1 in the presence of m1 or m2 antisense RNAs was confirmed after growing several heteroplasmid clones for 60 more generations in the absence of Tc (not shown). These results demonstrated that pairing with the *copG-repB* mRNA through the entire 5′-tail of antisense RNAII is required for *in vivo* inhibition of *repB* translation.

### Determination of the relative amount of antisense RNA in pneumococcal cells containing different pCGA9 plasmid variants

**Figure 5:**
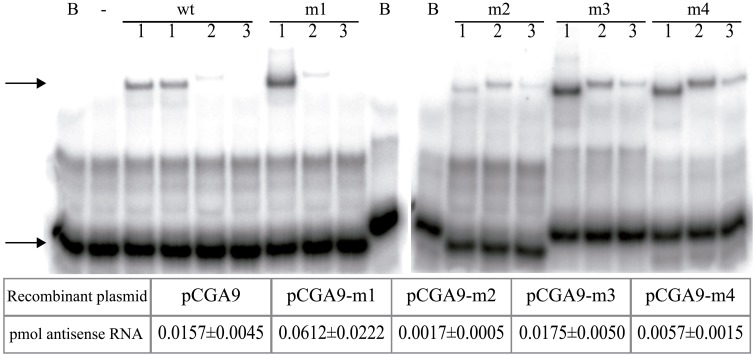
**Relative intracellular amounts of the antisense RNA variants**. Oligo-RNA band-shifts assays were performed with total RNA extracted from pneumococcal cells containing the different pCGA9 recombinant plasmid variants. Arrows point to the position of the free oligonucleotide (lower band) and that of the oligo-RNAII hybrids (upper band). B, sample lacking any RNA; -, sample containing *in vitro* synthesized mRNA_80_ and lacking antisense RNA;. Lane 1, samples containing 4 μg total RNA from cells that encode the indicated antisense RNA. Samples prepared with two independent extractions of RNAII wt are shown. Lanes 2 and 3, samples containing 0.025 and 0.0025 pmol, respectively, of the indicated *in vitro* synthesized antisense RNAs. The table below shows the amount of antisense RNA (pmol) per μg of total RNA. The values displayed are the mean of at least four independent assays.

Mutations in the *rnaII* gene may alter the turnover, and hence the intracellular concentration, of the mutant antisense RNAs. Since the intracellular concentration of RNAII has been shown to correlate with the level of incompatibility against the pMV158 replicon (del Solar et al., 1995), unbiased analysis of the *in vivo* inhibitory ability of the antisense RNA variants required estimation of the relative amount of these RNAs in pneumococcal cells containing the corresponding pCGA9 plasmid variant. To this end, “oligo-RNA band-shift” assays (Figure 5) were performed by using total RNA from each homoplasmid strain and a radioactively-labeled complementary oligo (BS, BS-m3, or BS-m4, see Materials and Methods). As controls, known amounts of the *in vitro* synthesized antisense RNAs were employed in these experiments. Table in Figure 5 shows the estimated amount of antisense RNA per μg of total RNA in cells bearing each recombinant pCGA9 variant. Only the mutation affecting the hairpin loop seemed not to alter significantly the turnover of the antisense RNA, as the intracellular concentration of the m3 mutant was similar to that of the wt RNA. Mutation in the 5′-end (region-1) resulted in a 4-Fold increase of the intracellular amount of the antisense RNA, whereas mutation in region-2 or in the upper stem (region-4) of RNAII caused a 9- or 3-Fold decrease, respectively, in the relative concentration of the mutant antisense RNAs.

## Discussion

In this communication we report the analysis of the RNAII-mRNA interaction pathway leading to the formation of the inhibitory complex that controls replication of plasmid pMV158. As highlighted in a recent review, RNAII is the first and still only extrachromosomal element-encoded antisense RNA characterized in pneumococci (Wilton et al., 2015). Moreover, RNAII and, by extension, the equivalent RNAs encoded by plasmids of the pMV158 replicon family are among the smallest and simplest bacterial antisense RNAs described so far (Thomason and Storz, 2010). Hence, a detailed study on the pMV158 antisense control system can help to understand the mechanisms by which these small RNAs cope with base-pairing with, and regulation of, their target RNAs. Here we have analyzed the role of different regions and secondary structure elements of pMV158 RNAII in the binding to the target RNA and in the inhibition of *repB* translation. To this end, we mutated the nucleotide sequence of several elements of RNAII (Figure 1C) that might be involved in the recognition of the target mRNA, namely each of the two 8-nt-long halves of the 5′-tail (region-1 and region-2), as well as the apical loop (region-3) and the upper stem region (region-4) of the hairpin. As we wanted to analyze the effect of these changes on the *in vitro* binding to the mRNA and on *in vitro* and *in vivo* inhibition of *repB* expression, preservation of a functional intrinsic terminator (a stable hairpin followed by a 3′ U-tract), was a goal. Therefore, investigation on the participation of the RNAII 3′ U-tail is missing in the present work, although no indications of a main role of the equivalent region have been reported for other antisense RNA systems (Wagner and Simons, 1994; Cervantes-Rivera et al., 2010). Our results show that binding to mRNA involves mainly the RNAII region-2, located adjacent to the single hairpin of the antisense RNA (Figure 1C). In addition, pairing of the 5′-terminal region-1 of RNAII with its complementary sequence in the mRNA is shown to be essential for the efficient inhibition of *repB* translation initiation *in vitro* and *in vivo*. Taken together, these data reveal that the entire 5′-tail of RNAII plays a dual critical role, being required both for the RNAII/mRNA binding and for the mechanism of *repB* translation inhibition.

*In vitro* analysis of the interaction between antisense RNAII and its target region in mRNA_80_ yields binding-rate constants (Table 2) that are well within the range reported for other antisense-RNA control systems (1–50 × 10^5^ M^−1^ s^−1^; Wagner and Simons, 1994; Wagner and Brantl, 1998; Franch et al., 1999). These studies also show that region-2 of RNAII has a critical role in the RNAII/mRNA binding, as indicated by the fact that antisense RNA m2 binds to the mRNA by far with the lowest efficiency (Figure 2 and Table 2). In contrast, intermolecular pairing through the upper stem region of RNAII is dispensable for the rapid binding to the mRNA, whereas the loop and the 5′-terminal region-1 contribute moderately to the binding (Figure 2 and Table 2). Therefore, only single-stranded regions of RNAII seem to have a significant role in initiating binding to the target mRNA, as was expected.

Based on these data, we propose that binding of the antisense/sense RNAs of the pMV158 replication control system initiates by pairing of the single-stranded 5′-tail of RNAII with its complementary sequence in the mRNA, and is facilitated by formation of a “kissing complex” between antisense/sense cognate hairpin loops. Moreover, progression of the “kissing” pairing into the upper stems of the complementary hairpins is not required for efficient formation of an RNAII/mRNA stable complex. This antisense/sense RNA interaction pathway based on a linear-linear scheme contrasts with the loop-loop model proved for other plasmid replication control systems, where the “kissing” interaction is crucial for binding of both RNAs, and the initial loop/loop base-pairing is propagated into the upper part (as in R1) or even into the lower portion (as in pIP501) of the stems (Hjalt and Wagner, 1995; Wagner and Brantl, 1998; Kolb et al., 2000; Heidrich and Brantl, 2007). Mutations in the recognition loops of the antisense RNAs of these well-known systems lead to new incompatibility groups (Tomizawa and Itoh, 1981; Wagner and Simons, 1994; Brantl and Wagner, 1996), which highlight the importance of the “kissing” interaction for RNA-RNA binding. Contrarily, changing the entire sequence of the apical loop of the single RNAII hairpin does not abolish the ability of the mutant antisense RNA (RNA m3) to produce incompatibility against pMV158 (Figure 4). In fact, only changes in the 5′-tail of RNAII (RNAs m1 and m2) suppress the inhibitory activity of the antisense RNA and are, therefore, predicted to lead to new incompatibility groups (Figure 4). The rather secondary role played by the apical loop of pMV158 RNAII (and hence by the corresponding “kissing”) in antisense/sense RNA binding is consistent with previous observations that *repB* mRNA variants lacking the complementary target hairpin were still susceptible to RNAII-mediated inhibition of *repB* translation (del Solar and Espinosa, 1992). These results led to the suggestion that the pMV158 system was unique among the plasmid replication control systems mediated by antisense RNA in that the initial interaction with the mRNA was based in base-pairing through the 5′-tail of RNAII (del Solar and Espinosa, 1992; Franch et al., 1999). In the linear-linear model proposed here, the entire 5′-tail and the hairpin loop of RNAII would participate in the recognition of the mRNA, although the 3′ half of the 5′-tail would play a further critical role in formation of a stable complex. Dispensability of the putative “kissing complex” in the pMV158 system has been related to the lack of U-turn motifs in the complementary hairpin loops of the inhibitor and target RNAs (Kim et al., 2008). It is worth noting that, although initiation of the RNAII/mRNA binding pathway seems to differ notably from the general U-turn-dependent “kissing” reported for other plasmid replication antisense-control systems, the RNA/RNA association rate constant obtained for the pMV158 system is in the same order of magnitude as those reported for the other plasmids. Thereby, the lack of a U-turn motif in both of the complementary hairpin loops of the pMV158 antisense/sense RNAs appears to be overcome by an efficient bi-partite interaction involving both the hairpin loop and the single-stranded 5′-tail of RNAII, as well as their corresponding complementary sequences in the mRNA.

The fact that sequence substitution of region-2 impairs binding to the mRNA much more severely than substitution of region-1 is rather intriguingly as both RNAII regions share features theoretically related to the efficiency of interaction with the target RNA (Matveeva et al., 2003; Shao et al., 2006). Namely, region-1 and region-2 are located in the 5′-tail of RNAII and have the same high A-U content (6 A/U out of 8 nt), and hence they would have been expected to contribute similarly to RNAII/mRNA binding. It should be noted that the RNA/RNA binding experiments performed in this work do only allow the observation of complexes persistent to gel electrophoresis. Hence, the extraordinaire impairment in binding efficiency observed for antisense RNA m2 might arise from the participation of region-2 not only in the initial interaction with the mRNA, but also in a subsequent step of intermolecular pairing progression leading to the formation of a stable complex.

We have previously proposed that the different folding of mRNA_60_ and mRNA_80_ (with the latter exposing the entire sequence complementary to the 5′-tail of RNAII as single strand) might account for the ~2-3-Fold higher efficiency of binding of the antisense RNA to mRNA_80_ relative to mRNA_60_ (López-Aguilar and del Solar, 2013). The differential binding of RNAII to mRNA_60_ and mRNA_80_ suggests that the efficiency of the RNAII/mRNA interaction might vary as the structure of the nascent mRNA changes. However, timing does not seem to be critical for RNAII-mediated *repB* translation inhibition, since this occurs efficiently irrespective of whether RNAII binds to growing mRNA molecules (*in vivo* incompatibility tests) or to full-length *copG-repB* mRNA (*in vitro* translation experiments). These findings contrast with those reported for the antisense control system of ColE1, where inhibitory binding of the antisense RNA occurs when nascent pre-primer RNA is 100-150 nt long. Once the pre-primer has grown longer, binding of the antisense RNA is still possible, but it does not prevent folding of the pre-primer RNA into the set of secondary and tertiary structures that are crucial for primer maturation (Masukata and Tomizawa, 1986; Wagner and Simons, 1994). The existence of a time window for inhibition of ColE1 replication by the antisense RNA arises from the indirect control mechanism used in this system, where pairing between the antisense and target RNAs blocks the formation of essential distal structures in the untranslated pre-primer RNA. On the contrary, the apparent lack of a time window for inhibition mediated by the pMV158 antisense RNA is consistent with direct hindrance of the access of the ribosomes to the *repB* translation initiation signals upon binding of RNAII to its complementary region in the mRNA. Although the RNAII target does not include actually any sequence element required for efficient *repB* translation, formation of a stable RNA-RNA duplex may prevent binding of the ribosomes to the immediately downstream region and hence expression of the essential gene (López-Aguilar et al., 2013). Moreover, the tight coupling of transcription and translation in prokaryotes likely constrains the folding of the mRNA within local regions (Shao et al., 2006), so that the structure of the target of RNAII, which lies entirely in the *copG-repB* intergenic portion of the mRNA, may remain rather invariable throughout the biosynthesis of the messenger.

Inhibition of *repB* translation by RNAII does not require formation of an antisense RNA/mRNA complete duplex, since swapping the complementary bases at the upper stem of the RNAII hairpin hardly decreases the inhibitory ability of the antisense RNA *in vivo* or *in vitro* (Figures 3, 4). In contrast, efficient inhibition by RNAII depends on pairing to the target mRNA through the 5′-tail of the antisense RNA.

Since stable binding to its target mRNA might be a prerequisite for RNAII to inhibit *repB* translation, we cannot rule out that the almost null inhibitory ability of antisense RNA m2 results solely from the severe impairment that substitution of region-2 causes on the efficiency of binding to the mRNA (Figures 2–4 and Table 2). Therefore, no information on the participation of region-2 in the mechanism of inhibition itself could be inferred from either the *in vivo* incompatibility tests or the *in vitro* translation inhibition assays. Contrarily, region-1 of RNAII is shown to have a main role in the mechanism of inhibition of *repB* translation since antisense RNA m1, while still retaining a moderate efficiency of binding to the mRNA (Figures 2–4 and Table 2), lacks the ability to produce incompatibility against pMV158 (Figure 4) and only slightly reduces *repB* translation (Figure 3). The failure of antisense RNA m1 to inhibit *in vivo* replication of pMV158 occurs despite its increased intracellular concentration, which is 4 times as high as that of the wt RNAII (Figure 5). On the other hand, base-pairing with the mRNA target through the apical loop and upper stem of the RNAII hairpin does not seem to contribute to the inhibitory mechanism itself. Although, compared to the wt RNAII, the m3 mutant shows a 5-Fold decreased efficiency of binding to the mRNA, it still causes a significant level of incompatibility against pMV158 and reduces *repB* translation by ~20-Fold. Swapping the base pairs at the upper stem hardly impaired the binding efficiency of the antisense RNA or its ability to inhibit *repB* translation *in vivo* or *in vitro* (Figures 2–4 and Table 2). The fact that inhibition of *repB* expression requires intermolecular pairing through region-1 of RNAII is consistent with the target of this region on the mRNA lying just upstream of the sequences involved in binding of the ribosomes (Figure 1B; López-Aguilar et al., 2013). Hence, whenever the antisense RNA/mRNA complex, although efficiently formed, allows the access of the ribosomes to the *repB* translation initiation signals, inhibition is avoided and synthesis of the plasmid replication initiator protein takes place.

## Concluding remarks

Taking into account the inhibition and binding results obtained with the antisense RNA variants, we can conclude that the 5′-tail of RNAII plays critical roles in both processes. The 3′ half of this tail is required for generation of a stable RNA-RNA complex, predictably because its location adjacent to the RNAII hairpin renders this region essential for propagation of intermolecular pairing into the G-C rich sequence of the antisense RNA. On the other hand, the 5′ half of the tail is essential for inhibiting *repB* translation.

The pMV158 antisense system studied in the present work is singular among the plasmid replication control systems in that: (i) antisense RNAII (48-nt long) is the smallest characterized so far; (ii) contrarily to other systems that also involve pairing of complementary sequences located on cognate secondary structure elements of the inhibitor and target RNAs, formation and progression of a “kissing complex” is not a critical step in this plasmid; (iii) efficient binding to the mRNA requires intermolecular pairing through the RNAII region-2 5′-adjacent to the hairpin; (iv) in contrast to what has been reported for other systems (Wagner and Simons, 1994), efficient RNA-RNA binding does not ensure efficient *in vivo* or *in vitro* inhibition of *repB* translation; instead, two distinct regions of the 5′-tail of RNAII have critical roles in binding to the mRNA target and in preventing *repB* translation.

### Conflict of interest statement

The authors declare that the research was conducted in the absence of any commercial or financial relationships that could be construed as a potential conflict of interest.

## Footnotes

^1^http://mfold.rna.albany.edu
