# Supplementary material for: The 5′-tail of antisense RNAII of pMV158 plays a critical role in binding to the target mRNA and in translation inhibition of repB
Source: Front Genet. 2015 Jun 30;6:225. doi: 10.3389/fgene.2015.00225 (PMC4485353; doi:10.3389/fgene.2015.00225)
Supplement: Supplementary file 1 [file Table1.DOCX]

***Supplementary Material***

The 5’-tail of antisense RNAII of pMV158 plays a critical role in binding to the target mRNA and in translation inhibition of *repB*

Celeste López-Aguilar^1^, Cristina Romero-López^2^, Manuel Espinosa^1^, Alfredo Berzal-Herranz^2^, Gloria del Solar^1^

^1^ Molecular Microbiology and Infection Biology Department, Centro de Investigaciones Biológicas (CIB-CSIC), Madrid, Spain

^2^ Molecular Biology Department, Instituto de Parasitología y Biomedicina López-Neyra (IPBLN-CSIC), Granada, Spain.

*Correspondence: Gloria del Solar, CIB, CSIC, Ramiro de Maeztu, 9, 28040 Madrid, Spain. [**gdelsolar@cib.csic.es**](mailto:gdelsolar@cib.csic.es)

1. **Supplementary Table** **1. List of oligonucleotides used in this work**

| Name | Size  (nt) | Sequence 5’- 3’ (in pMV158) | Use for |
| --- | --- | --- | --- |
| pC 1800-1818 | 19 | ccgctatctttacaggtac | PCR |
| pC 2798-2780 | 19 | tgatagacgaaatcgagga | PCR |
| F-RNAII-m1 | 48 | cactggctaaagtcaactccggaatgggtatattatactttatggcta | mutagenesis |
| R-RNAII-m1 | 48 | tagccataaagtataatatacccattccggagttgactttagccagtg | mutagenesis |
| F-RNAII-m2 | 50 | gcaggcactggctccccatggacatttcttgggtatattatactttatgg | mutagenesis |
| R-RNAII-m2 | 50 | ccataaagtataatatacccaagaaatgtccatggggagccagtgcctgc | mutagenesis |
| F-RNAII-m3 | 48 | tcaagaaaaataaaaaaagccgtggatcgccccactggctaaagtcaa | mutagenesis |
| R-RNAII-m3 | 48 | ttgactttagccagtggggcgatccacggctttttttatttttcttga | mutagenesis |
| F-RNAII-m4 | 48 | tcaagaaaaataaaaaaagcccacgtggcagcgtgtggctaaagtcaa | mutagenesis |
| R-RNAII-m4 | 48 | ttgactttagccacacgctgccacgtgggctttttttatttttcttga | mutagenesis |
| RNAII | 83 | ataaaaaaagccgtgctggcaggcactggctaaagtcaaacatttcttcctatagtgagtcgtattacaattctccggcctcc | *in vitro* transcription |
| RNAII-m1 | 83 | ataaaaaaagccgtgctggcaggcactggctaaagtcaactccggaatcctatagtgagtcgtattacaattctccggcctcc | *in vitro* transcription |
| RNAII-m2 | 83 | ataaaaaaagccgtgctggcaggcactggctccccatggacatttcttcctatagtgagtcgtattacaattctccggcctcc | *in vitro* transcription |
| RNAII-m3 | 83 | ataaaaaaagccgtggatcgccccactggctaaagtcaaacatttcttcctatagtgagtcgtattacaattctccggcctcc | *in vitro* transcription |
| RNAII-m4 | 83 | ataaaaaaagcccacgtggcagcgtgtggctaaagtcaaacatttcttcctatagtgagtcgtattacaattctccggcctcc | *in vitro* transcription |
| P-RNAII | 23 | ggaggccggagaattgtaatacg | *in vitro* transcription |
| P2-RNAII | 21 | ataaaaaaagccgtgctggca | *in vitro* transcription |
| P3-RNAII | 21 | ataaaaaaagccgtggatcgc | *in vitro* transcription |
| P4-RNAII | 22 | ataaaaaaagcccacgtggcag | *in vitro* transcription |
| mRNA | 61 | ggaggccggagaattgtaatacgactcactataggataaaaaaagccgtgctggcaggcac | *in vitro* transcription |
| mRNA-80 | 71 | ctttttctttagccataaagtataatatacccaagaaatgtttgactttagccagtgcctgccagcacggc | *in vitro* transcription |
| mRNA-80R | 23 | ctttttctttagccataaagtat | *in vitro* transcription |
| BS | 17 | tgctggcaggcactggc | oligo band-shift |
| BS-m3 | 17 | tggatcgccccactggc | oligo band-shift |
| BS-m4 | 17 | acgtggcagcgtgtggc | oligo band-shift |
